# Recombination analysis on the receptor switching event of MERS-CoV and its close relatives: implications for the emergence of MERS-CoV

**DOI:** 10.1186/s12985-024-02358-2

**Published:** 2024-04-10

**Authors:** Jarel Elgin Tolentino, Spyros Lytras, Jumpei Ito, Kei Sato

**Affiliations:** 1 Division of Systems Virology, Department of Microbiology and Immunology, The Institute of Medical Science, The University of Tokyo, Tokyo, Japan; 2 Graduate School of Frontier Sciences, The University of Tokyo, Kashiwa, Japan; 3 MRC–University of Glasgow Centre for Virus Research, Glasgow, UK; 4 International Vaccine Design Center, The Institute of Medical Science, The University of Tokyo, Tokyo, Japan; 5 Graduate School of Medicine, The University of Tokyo, Tokyo, Japan; 6 CREST, Japan Science and Technology Agency, Kawaguchi, Japan; 7 International Research Center for Infectious Diseases, The Institute of Medical Science, The University of Tokyo, Tokyo, Japan; 8 Collaboration Unit for Infection, Joint Research Center for Human Retrovirus Infection, Kumamoto University, Kumamoto, Japan

**Keywords:** Coronavirus, MERS-CoV, Recombination, Receptor switching event, One health

## Abstract

**Background:**

PlMERS-CoV is a coronavirus known to cause severe disease in humans, taxonomically classified under the subgenus *Merbecovirus.* Recent findings showed that the close relatives of MERS-CoV infecting vespertillionid bats (family *Vespertillionidae*), named NeoCoV and PDF-2180, use their hosts’ ACE2 as their entry receptor, unlike the DPP4 receptor usage of MERS-CoV. Previous research suggests that this difference in receptor usage between these related viruses is a result of recombination. However, the precise location of the recombination breakpoints and the details of the recombination event leading to the change of receptor usage remain unclear.

**Methods:**

We used maximum likelihood-based phylogenetics and genetic similarity comparisons to characterise the evolutionary history of all complete *Merbecovirus* genome sequences. Recombination events were detected by multiple computational methods implemented in the recombination detection program. To verify the influence of recombination, we inferred the phylogenetic relation of the merbecovirus genomes excluding recombinant segments and that of the viruses’ receptor binding domains and examined the level of congruency between the phylogenies. Finally, the geographic distribution of the genomes was inspected to identify the possible location where the recombination event occurred.

**Results:**

Similarity plot analysis and the recombination-partitioned phylogenetic inference showed that MERS-CoV is highly similar to NeoCoV (and PDF-2180) across its whole genome except for the spike-encoding region. This is confirmed to be due to recombination by confidently detecting a recombination event between the proximal ancestor of MERS-CoV and a currently unsampled merbecovirus clade. Notably, the upstream recombination breakpoint was detected in the N-terminal domain and the downstream breakpoint at the S2 subunit of spike, indicating that the acquired recombined fragment includes the receptor-binding domain. A tanglegram comparison further confirmed that the receptor binding domain-encoding region of MERS-CoV was acquired via recombination. Geographic mapping analysis on sampling sites suggests the possibility that the recombination event occurred in Africa.

**Conclusion:**

Together, our results suggest that recombination can lead to receptor switching of merbecoviruses during circulation in bats. These results are useful for future epidemiological assessments and surveillance to understand the spillover risk of bat coronaviruses to the human population.

**Supplementary Information:**

The online version contains supplementary material available at 10.1186/s12985-024-02358-2.

## Introduction

Coronaviruses (CoVs) are a group of RNA viruses known to cause infectious diseases in mammals, including humans [1]. To date, all human CoVs (HCoV) belongs to two genera: (1) *Alphacoronavirus*—including subgenera *Duvinacovirus* (HCoV-229E), and *Setracovirus* (HCoV-NL63), and (2) *Betacoronavirus*—including subgenus *Embecovirus* (HCoV-HKU1), subgenera *Sarbecovirus* (Severe Acute Respiratory Syndrome CoV [SARS-CoV], SARS-CoV-2), and *Merbecovirus* (Middle East Respiratory Syndrome CoV [MERS-CoV]) [2, 3]. All these HCoVs are divided based on their pathogenicity and symptoms presentation, the four seasonal HCoVs (HCoV-OC43, HCoV-229E, HCoV-NL63, and HCoV-HKU1) typically result in mild to moderate respiratory symptoms; and the three others—MERS-CoV, SARS-CoV, and SARS-CoV-2—are known to cause more severe symptoms [2, 4]. MERS-CoV, SARS-CoV, and SARS-CoV-2 have been recognized as diseases of high priority by the World Health Organization (WHO) [5], with SARS-CoV-2 being responsible for the COVID-19 pandemic in 2020 [6].

HCoVs are believed to have emerged through spillover events, where animal CoVs transmitted to humans [7]. For example, SARS-CoV and SARS-CoV-2 are considered to have originated from bat coronaviruses according to the evidence that coronaviruses closely related to SARS-CoV and SARS-CoV-2 are circulating within *Rhinolophus* bats living around East and Southeast Asia [8, 9]. For another instance, MERS-CoV infection in humans is zoonotically sourced from camel hosts [10], but more research shows that the virus originated from bats because of its phylogenetic proximity to a wide group of bat-borne coronaviruses [11, 12]. Some merbecoviruses such as HKU4 and HKU5 have been exclusively sampled in vespertilionid bats (family *Vespertilionidae*) which are distributed worldwide except for the Arctic and Antarctica continents [13, 14]. Understanding these spillover events is crucial, which involves identifying: (i) the types of viruses in the wildlife that have the potential to infect humans, and (ii) the mechanisms through which these viruses adapt to gain human infectivity.

Recently, close relatives of MERS-CoV have also been identified in vespertilionid bats in Africa and named NeoCoV and PDF-2180 [11, 12]. NeoCoV is the closest relative of MERS-CoV with an 85.5–85.6% whole-genome nucleotide sequence identity [11]. Importantly, although MERS-CoV utilizes dipeptidyl peptidase 4 (DPP4) as the viral receptor [15], a previous study by Xiong et al. (2022) showed that NeoCoV and PDF-2180 utilize angiotensin-converting enzyme 2 (ACE2) as their viral receptor [16]. Additionally, another study has identified bat coronaviruses related to MERS-CoV that also use ACE2 as their viral receptor [17]. These findings suggest that receptor usage of these coronaviruses switched from ACE2 to DPP4 (or from DPP4 to ACE2) during their recent evolution.

Coronaviruses enter the cell through a binding interaction between the spike glycoprotein on the viral envelope and the viral receptor on the cellular membrane [18]. The virus receptor usage is thus determined by the spike protein, particularly by the spike receptor binding domain (RBD) [19]. Consequently, amino acid changes in the spike protein, particularly the RBD, can lead to switching the receptor usage of a coronavirus [20]. Supporting this, Xiong et al. (2022) demonstrated that although NeoCoV and PDF-2180 have high overall nucleotide similarity to MERS-CoV, this similarity decreases significantly in the region of the spike gene, especially in the region including the RBD-encoding region [16]. This finding suggests that significant evolutionary changes took place in the spike protein sequence of either the common ancestor of MERS-CoV or that of NeoCoV and PDF-2180, which led to the switch in their receptor usage.

Recombination among distinct viruses can profoundly impact their phenotypes because it allows the simultaneous change of entire regions of the genome in a single evolutionary event [21]. For example, animal retroviruses like murine leukemia virus and feline leukemia virus occasionally change their receptor usage through recombination within an infected host individual [22, 23]. Importantly, coronaviruses are known to undergo frequent recombination throughout their evolutionary history [24]. Xiong et al. (2022) propose that the receptor switch in the common ancestor of MERS-CoV or that of NeoCoV and PDF-2180 may have been caused by recombination, resulting in the exchange of (a part of) the spike gene [16]. However, previous studies on the topic lacked comprehensive genetic and phylogenetic analyses to reveal the evolutionary trajectory of these viruses including the occurrence of recombination. Specifically, it remains unclear (i) to what extent recombination has influenced the evolution of the merbecoviruses, (ii) the exact range of the genetic region acquired by recombination, and (iii) the evolutionary direction of the receptor switch (whether the ancestral state virus was an ACE2-user like NeoCoV or a DPP4-user like MERS-CoV). To address these questions, we performed detailed recombination and phylogenetic analyses, aiming to illustrate the evolutionary trajectory of merbecoviruses leading to the emergence of MERS-CoV.

## Methods

### Data acquisition

The complete genome sequences of all merbecoviruses were obtained as of the 10th of October 2023 from NCBI using Nucleotide Advanced Search Builder (https://www.ncbi.nlm.nih.gov/nuccore/advanced) with the following search queries with an ‘OR’ condition (*Merbecovirus*, Middle East respiratory syndrome-related coronavirus, *Tylonycteris* bat coronavirus HKU4, Bat MERS-like coronavirus, *Pipistrellus* bat coronavirus HKU5, *Merbecovirus* sp., Hedgehog coronavirus 1, *Erinaceus* hedgehog coronavirus HKU31, *Hypsugo* bat coronavirus HKU25, *Pipistrellus* abramus bat coronavirus HKU5-related, *Tylonycteris pachypus* bat coronavirus HKU4-related, *Merbecovirus* sp. PaGB01, Betacoronavirus sp. isolate BtCoV/P.nathusii/NL/2018 − 403.3, and Bat coronavirus isolate PREDICT/PDF-2180). The search queries were selected according to the grouping in NCBI Taxonomy Browser [25] of the subgenus *Merbecovirus* (taxid 2509494). From each designated species or isolate (except for human MERS-CoV) under the subgenus we downloaded all genome entries available in the taxonomy browser. Due to the over-representation of human and camel MERS-CoV sequences, we selected a subset of genomes as follows: (i) for MERS-CoV clades A and C we selected two complete genomes out of each clade, ensuring that one of the two clade A genomes is the NCBI reference genome (isolate HCoV-EMC/2012, accession: NC_019843.3); (ii) MERS-CoV clade B is separated in seven lineages and to capture this diversity in our analysis we selected two complete genomes out of each lineage. To capture more diversity of clade B, an additional 2 sequences sampled in other countries besides the Arabian/African continent were added (a total of 16 clade B genomes). Two additional, recently published, bat merbecovirus genomes (isolates MOW15-22 and PnNL2018B, with accessions ON325306.1 and OQ405399.1 respectively) were not grouped under the *Merbecovirus* subgenus in the taxonomy browser, but were included in our dataset. This led to a final dataset of 49 complete merbecovirus genomes (Table S1). As an outgroup, four representative sarbecovirus genomes, SARS-CoV (SZ3: AY304486.1, TOR2: NC_004718.3), SARS-CoV-2 (Wuhan-Hu-1: NC_045512.2) and RaTG13 (MN996532.2), were further used in some analyses (described below in detail).

### Phylogenetic analysis

**Fig. 1:**
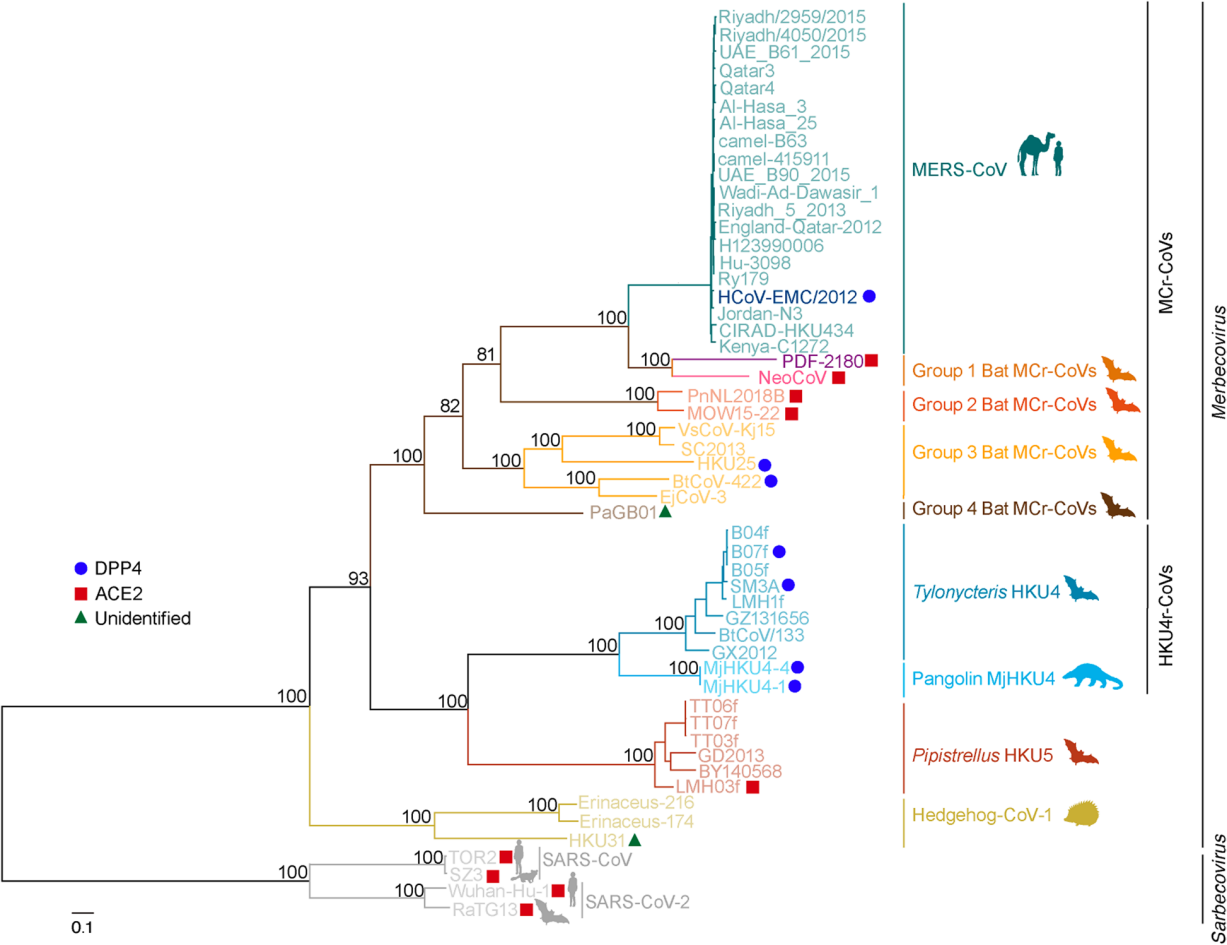
Maximum-likelihood phylogenetic tree based on the complete genome of the merbecoviruses. The red squares represent ACE2 usage, blue circles represent DPP4 usage, and green triangles represent unidentified receptor usage. Numerical node support values are shown above each internal node. The scale bar represents genetic distance. Four representative ACE2-using sarbecoviruses were used for rooting the phylogeny

**Fig. 2:**
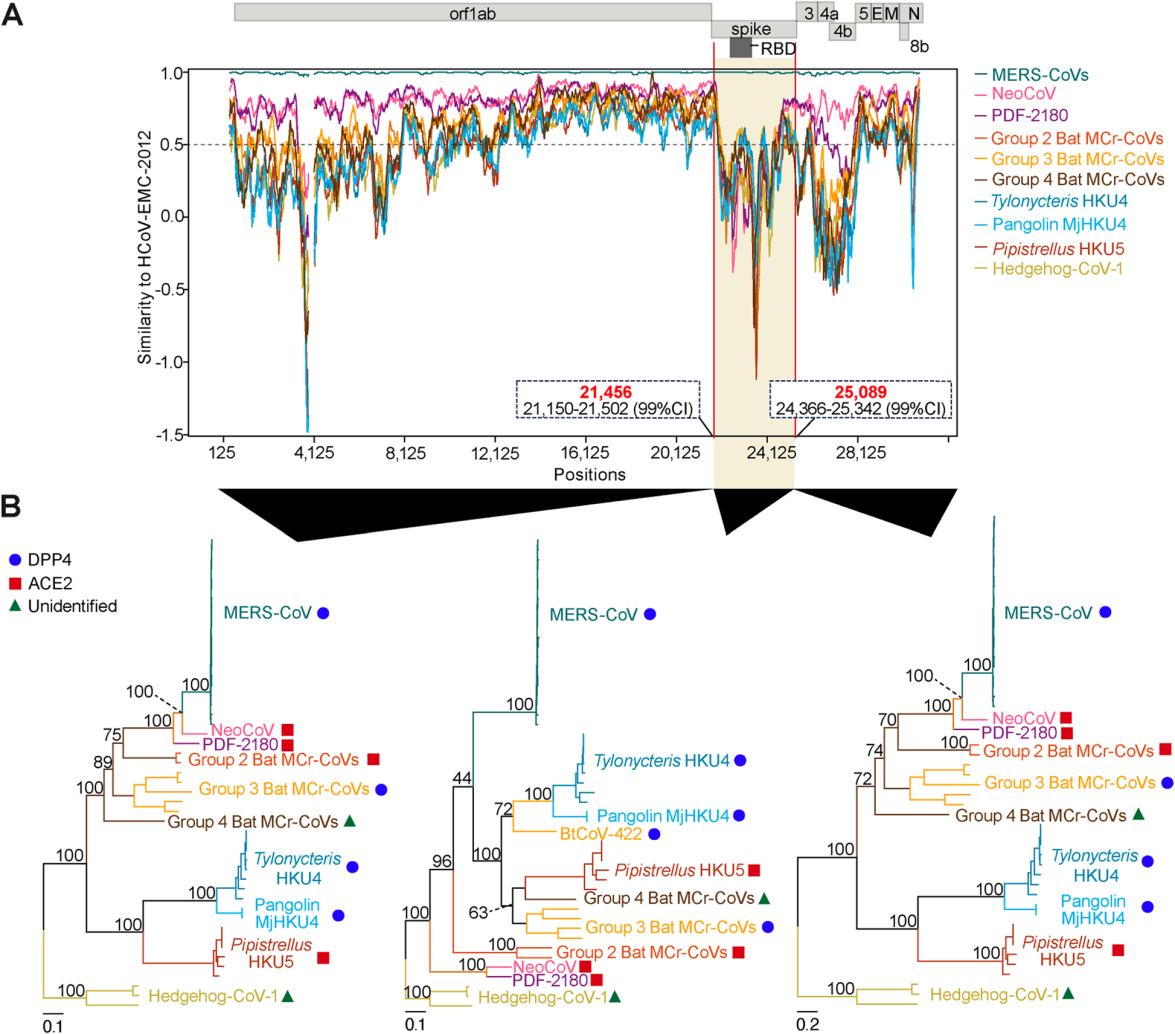
Sequence similarity across *Merbecovirus* genomes.  **A**) HCoV-EMC-2012 was used as the query reference. All MERS-CoV representative isolates from clades A, B and C were converted to a consensus sequence referred to as “MERS-CoVs” in the plot. PDF-2180 and NeoCoV were separated as they were the most closely related virus to MERS-CoVs. PnNL2018B and MOW15-22 were converted to consensus and grouped into “group 2 Bat MCr-CoVs”. SC2013, HKU25, VsCoV-kj15, EjCoV-3 and BtCoV-422 and were converted to consensus into “group 3 Bat MCr-CoVs”. PaGB01 was named into “group 4 Bat MCr-CoVs”. SM3A, GZ131656, BtCoV/133, B04f, B05f, B07f, LMH1f and GX2012 were converted to consensus into “ Tylonycteris HKU4”. MjHKU4-1 and MjHKU4-4 were converted to consensus into “Pangolin MjHKU4”. LMH03f, TT03f, TT06f, TT07f, GD2013 and BY140568 were converted to consensus into “ Pipistrellus HKU5”. Erinaceus-174, Erinaceus-216, and HKU31 were converted to consensus into “Hedgehog-CoV-1”. The groupings were based on the NCBI Taxonomy browser [25]. The x-axis represents the similarity score, while the y-axis represents the genomic coordinates.  **B**) Maximum likelihood phylogenetic trees inferred based on the fragmented regions of MERS-CoV. This was based on the recombination breakpoints detected at position 21,456 in the NTD and 25,089 in the S2 of the spike region. All trees were rooted by the hedgehog-CoVs clade, consistent with Fig. 1. The red squares represent ACE2 usage, blue circles represent DPP4 usage, and green triangles represent unidentified receptor usage. The numerical node support values are shown above each node. The scale bars represent genetic distance

The obtained sequences were aligned in MAFFT v.7.520 [26] using default parameters. The aligned sequences were cleaned by replacing any characters not in ‘ATGCN-’ with ‘N’ using an in-house python script. Alignment sites with more than 10% of sequences containing poorly aligned or illegitimate regions were trimmed using trimAl v.1.2 [27]. A maximum likelihood (ML) phylogenetic tree of the genome was inferred by IQTree v.2.2.2.6 under a general-time reversible (GTR) nucleotide substitution model [28]. Node support was computed using ultrafast bootstraps with 1000 replicates [29]. In Fig. 1, complete genome sequences of merbecoviruses and sarbecoviruses were used. In Fig. 2B, the complete genome alignment of the merbecoviruses was fragmented based on the recombination breakpoints detected in MERS-CoV (positions 21,456 and 25,089), and then the phylogenetic tree for each fragment was reconstructed as described above. All tree visualizations were generated using ggtree v.3.8.2 package in R v.4.3.1.

### Similarity plot analysis

The complete genome sequences of merbecoviruses were analyzed based on their nucleotide similarities to the MERS-CoV representative genome (HCoV-EMC/2012). Similarity was plotted using SimPlot + + analysis with the Kimura-2-parameter model, a 250 bp sliding window and a step size of 20 bp [30].

### Recombination analysis

The representative complete genome sequence data (see Table S1) was screened for recombination using the Recombination Detection Program (RDP) v.5.44 (RDP5) [31] with the following statistical test methods: RDP [32], GENECONV [33], Chimaera [34], MaxChi [35], Bootscan [36], SiScan [37], and 3Seq [38]. Virus sequences were assumed to be linear and those with substantial signals for recombination by at least six methods out of seven were investigated further. The p-value threshold was set at 0.05 for all methods. These criteria were set to ensure that only confidently detected recombination events were selected [39, 40]. This led to the detection of 22 recombination events (Table S2). Next, we used BURT graphs paired with RDP, RESCAN, SISCAN, and DISTANCE plots, all implemented in RDP5, to validate the exact breakpoint positions of each recombination event in the genomes [31]. Next, we checked the consensus recombinant scores and validated if the detected recombinant has a weighted consensus score higher than its major and minor parents. This led to the detection of 18 recombination events (Table S2). To confirm the results of this recombination analysis, the beginning and the end of the breakpoints identified on the MERS-CoV genome were used to fragment the genome into non-recombinant regions (at least for MERS-CoV) for phylogenetic tree analysis as described above.

### Tanglegram analysis

Recombination-free sequences of the representative merbecoviruses were acquired using the RDP5 program. To get these sequences, we chose the option ‘save the alignments with recombinant regions removed’ after performing the recombination analysis described above. This method replaces all minor recombinant regions in the detected recombination events with gaps (‘-‘). On the other hand, the RBD-encoding region of the aligned *Merbecovirus*’ sequences were manually extracted using AliView v.1.28 [41]. The recombination-free tree and the RBD-encoding tree were compared using a tanglegram representation and visually examined for phylogenetic incongruences. The tanglegram analysis was performed using the phytools v.1.9–16 package in R v.4.3.1 [42]. The phylogenetic congruence between the two trees was assessed using the relative tree certainty metric implemented in RAxML v.8.2.12 [43, 44].

### Spatial distribution of virus sampling sites

Retrieval of the sampling location information was done by surveying previous papers where the representative merbecoviruses were described. The sampling location was mapped using city-level and are stated in Table S1. The map visualization was created using R v.4.3.1 with the following packages: tidyverse v.2.0.0, rnaturalearth v.1.0.1, and sf v.1.0–15.

## Results

Recombination has been suggested to contribute to the emergence in MERS-CoV [11, 12, 24, 45] but comprehensive genetic and phylogenetic analyses detailing this evolutionary mechanism have not been conducted yet. To confirm the recombination events leading to the emergence of MERS-CoV, we analyzed a set of 49 full-genome sequences of merbecoviruses (Table S1) and used it to reconstruct a maximum likelihood phylogeny. According to the tree topology, we classified merbecoviruses into 9 groups (Fig. 1): the MERS-CoV group that is a known human DPP4 (hDPP4)-using virus that infects camels and humans [15]; the group 1 bat MERS-related CoVs (MCr-CoVs) consisting of NeoCoV and PDF-2180 which were described to use bat ACE2 (bACE2) orthologues, and less efficiently human ACE2 (hACE2) [16]; the group 2 bat MCr-CoVs that includes the PnNL2018B and MOW15-22 that were recently documented to also use bACE2 [17]; the group 3 bat MCr-CoVs which includes bat viruses, like BtCoV-422 that can use hDPP4 and bDPP4 [46], and HKU25 that can use hDPP4 [47]; the group 4 bat MCr-CoVs that includes PaGB01 that cannot use hACE2, hDPP4 and human aminopeptidase N (hAPN) [48]; the *Tylonycteris* HKU4 group that uses hDPP4 and bDPP4 [49, 50]; the recently identified pangolin HKU4 that efficiently uses hDPP4, bDPP4, and pangolin DPP4 [51]; the *Pipistrellus* HKU5 group that cannot use hACE2, bACE2, hDPP4 or bDPP4 [16, 46, 49], but was recently shown to use *Pipistrellus abramus* ACE2 (the group’s reservoir bat host) [52], and lastly, the Hedgehog-CoV-1 group which also cannot use hACE2 or hedgehog ACE2 as its viral receptor [16]. The latter is the outgroup of the known merbecoviruses based on rooting by the sarbecoviruses (Fig. 1). Across their whole genome, the MERS-CoV sequences were found to be most closely related to the group 1 bat MCr-CoVs, as previously described [16, 51].

To detect the recombination events in merbecoviruses, we prepared a subset of the Fig. 1 dataset by removing redundant sequences from over-sampled *Merbecovirus* groups (Table S1). By using the recombination detection program (RDP5) [31], we confidently detected a total of 18 recombination events across the merbecoviruses using established and conservative criteria [31, 53, 54] (supported by 6 out of 7 methods implemented in RDP5 with a p-value cutoff of 0.05; see Methods and Table S2). This result suggests that multiple recombination events happened during the evolution of *Merbecovirus*. Of these 18 statistically-significant recombination events, one recombination event with two breakpoints, was detected in the spike region of MERS-CoV (HCoV-EMC/2012) [55]. The major recombinant parent was identified to be NeoCoV, while the minor recombinant parent was unknown, representing a likely yet unidentified merbecovirus clade. The starting recombination breakpoint was detected at position 21,456 of the N-terminal domain (NTD), while the ending recombination breakpoint was located at position 25,089 in the subunit 2 (S2) (coordinates corresponding to the reference MERS-CoV genome, HCoV-EMC/2012) (Table S3), encompassing the RBD (Fig. 2A). The genome regions upstream of the beginning breakpoint, and downstream of the ending breakpoint share 88% overall sequence identity between the MERS-CoV and NeoCoV genomes (see Fig. 2A). Contrastingly, the spike sequence of NeoCoV is highly different from that of MERS-CoV, aligned with previous reports [11, 16]. This result is consistent with the recombination findings.

To determine the phylogenetic placement of MERS-CoV relative to the other known merbecoviruses, we fragmented the genomic regions into three segments: (i) upstream of the first breakpoint (1–21,455), (ii) the recombinant region within the breakpoints (21,456 − 25,089), and (iii) downstream of the second breakpoint (25,090 − 30,119; positions corresponding to the HCoV-EMC/2012 genome). Based on the phylogenetic trees of (i) and (iii), MERS-CoV was confidently placed as the closest relative of NeoCoV (Fig. 2B). On the other hand, the phylogenetic tree of (ii) showed that the recombinant region of MERS-CoV did not form a group with NeoCoV and/or PDF-2180, but clustered within the known DPP4-using viruses such as *Tylonycteris* HKU4, Pangolin HKU4, BtCoV-422, and HKU25 (Fig. 2B). Together, this suggests that several recombination events took place in the recent evolution of the merbecoviruses, one of which describes the acquisition of a DPP4-using spike region by the NeoCoV-like proximal ancestor of MERS-CoV.

**Fig. 3:**
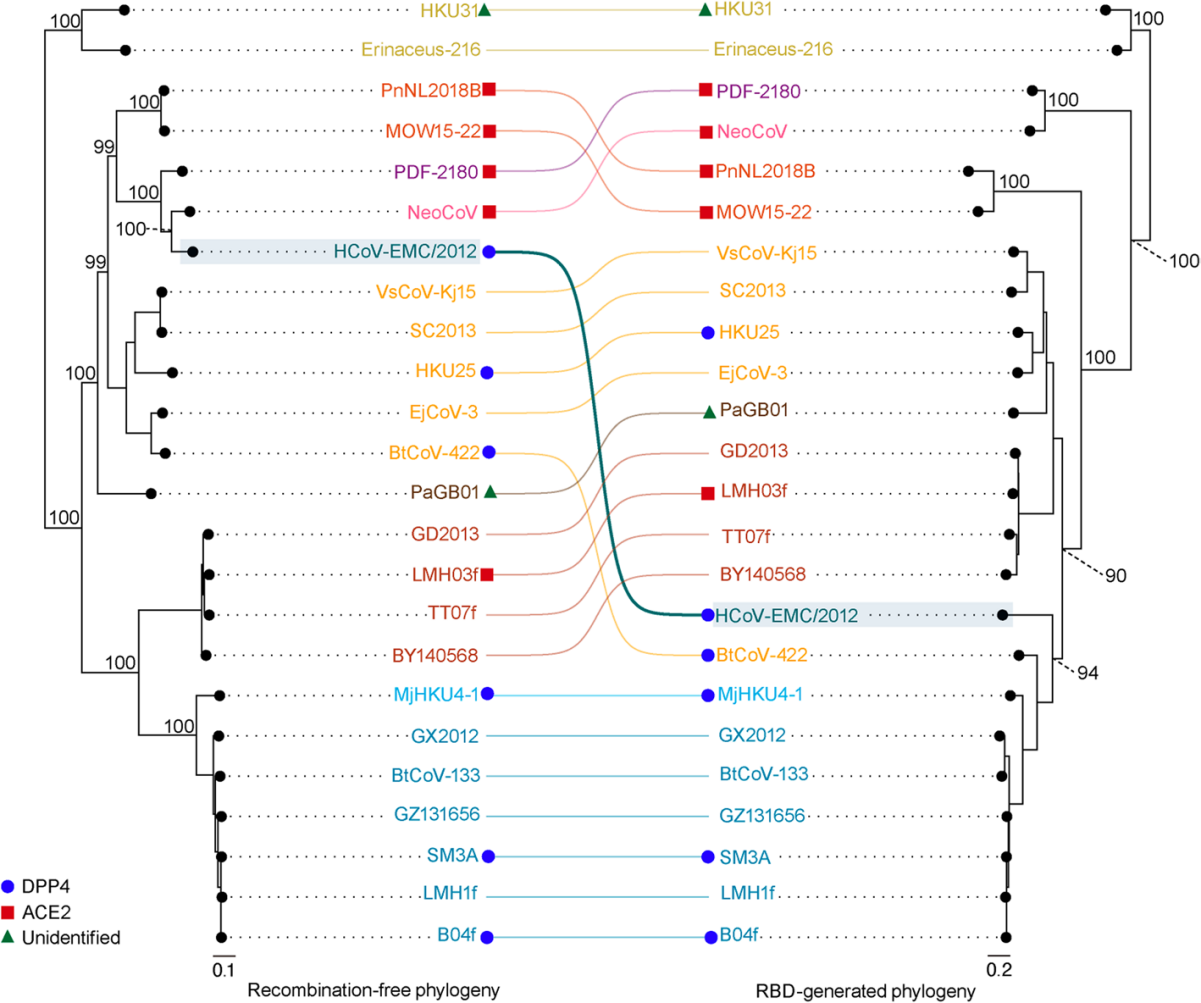
Comparison between the recombination-free and RBD merbecovirus phylogenies. Tanglegram of the representative merbecoviruses based on the comparison of the recombination-free phylogeny (left) and RBD-generated phylogeny (right). Highlighted in blue is the detected recombinant, HCoV-EMC/2012. The red squares represent ACE2 usage, blue circles represent DPP4 usage, and green triangles represent unidentified receptor usage. The numerical node support values are shown above each node. The scale bars represent genetic distance

To further verify the MERS-CoV recombination event, we generated a recombination-free alignment obtained from RDP5 (where minor recombinant regions are masked from the original multiple sequence alignment) and compared the resulting phylogeny to that of the viruses’ RBD tree (Fig. 3). We created a tanglegram to compare the topologies between these two trees and validate the effect of recombination on the MERS-CoV RBD. Less than half of the internal nodes between the two trees are congruent, having a relative tree certainty of 42.9% [44]. From the tanglegram visualisation it is evident that HCoV-EMC/2012 (the MERS-CoV reference genome) has a recombinant RBD region compared to the recombination-free rest of its genome. In the recombination-free phylogeny (reflecting most of the genome), HCoV-EMC/2012 confidently clusters next to NeoCoV, within group 1 (node support value: 100), while its RBD falls in a completely different place in the tree, close to those of the DPP4-using viruses (node support value: 94) (Fig. 3). Besides HCoV-EMC/2012, we also observed phylogenetic incongruence in BtCoV-422 indicating that this virus likely also has a recombinant RBD. Finally, the group 1 (PDF-2180 and NeoCoV) RBD falls more basally in the tree than the group’s recombination-free genome, indicating that the group may have experienced additional RBD switching, before the MERS-CoV ancestor acquired its DPP4-using RBD.

**Fig. 4:**
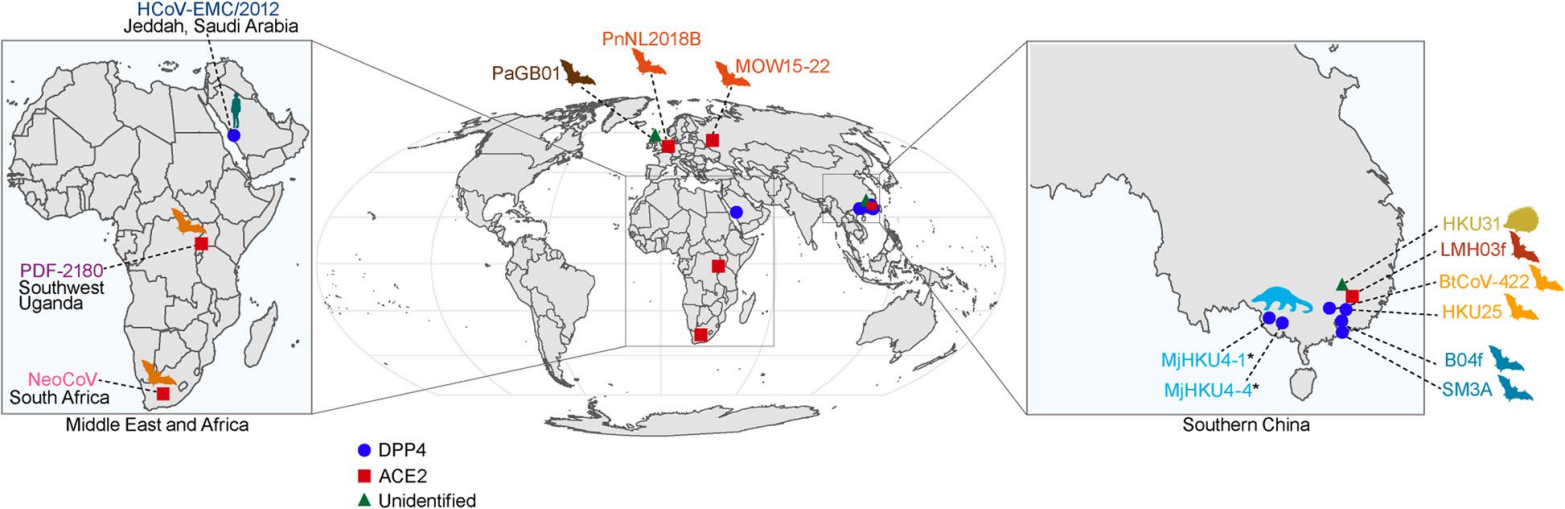
Geographical distribution of virus sampling sites. Global map showing the sampling location of all merbecoviruses with tested receptor usage. Location points on the map are labelled by the viruses’ receptor usage. The inset on the left highlights the emergence/sampling sites of HCoV-EMC/2012, PDF-2180 and NeoCoV (Middle East and Africa). The inset on the right highlights the sampling sites of HKU31, LMH03f, BtCoV-422, HKU25, B04f, and SM3A (Southern China). MjHKU4-1 and MjHKU4-4 (labelled with asterisks) sampled in pangolins (*Manis javanica*) were collected in Guangxi, China (as shown on the map), but the pangolins were originally retrieved during anti-smuggling events from Southeast Asia [51]

The presence of recombination implies that the non-recombinant proximal ancestor of MERS-CoV and the yet unsampled DPP4-using virus that gave MERS-CoV its present RBD must have co-circulated in the same host population at some point in the past. Hence, we decided to investigate the likely location of where this recombination event took place by examining the geographical distribution of the merbecovirus sampling sites (Fig. 4). The map shows that MERS-CoV (HCoV-EMC/2012) and the group 1 bat MCr-CoVs (NeoCoV and PDF-2180) were first found in quite distant locations in the Middle East and the African continent, respectively. Based on the current sampling, the proximal ancestor of MERS-CoV likely resided somewhere across the wide geographic region between Saudi Arabia and South Africa (Fig. 4, left map). The two newly documented ACE2-using merbecoviruses, PnNL2018B and MOW15-22 were sampled in Northern Europe (the Netherlands and Russia, respectively). Interestingly, all known animal DPP4-using merbecoviruses from bats and pangolins have been sampled in South China (Fig. 4, right map), indicating what seems to be a longitudinal separation between the most known ACE2-using (in Europe and Africa), and DPP4-using merbecoviruses (in Asia).

## Discussion

Our study confirms the previously suggested recombinant nature of the MERS-CoV spike [56–58] and sheds light on the precise recombination event that resulted in the DPP4-using MERS-CoV, first reported in humans in 2012. We show that MERS-CoV falls within the group 1 bat MCr-CoVs clade, containing NeoCoV and PDF-2180, for most of its genome (Fig. 3), however, unlike MERS-CoV, the two currently known group 1 viruses use the ACE2 receptor for cellular entry [16]. Our recombination analysis indicates that the NeoCoV-like, non-recombinant ancestor of MERS-CoV acquired a recombinant region between its spike NTD and S2 regions (genome positions 21,456 − 25,089) from a yet to be identified merbecovirus lineage – encompassing MERS-CoV’s present DPP4-using RBD (Fig. 2, Table S2). The tanglegram between the recombination-free tree (representing the undisturbed evolutionary history of the merbecoviruses) and the RBD tree illustrates the possible directionality of the recombination event (Fig. 3). Specifically, the MERS-CoV progenitor replaced its likely ACE2-using group 1 RBD with a DPP4-using RBD at some point after it diverged from NeoCoV (MERS-CoV’s closest known bat virus relative).

Recombination in RNA viruses has been documented extensively and is found to be more common in positive sense single-stranded RNA viruses including coronaviruses [59]. Recombination frequently occurs among related, co-circulating coronaviruses which leads to the emergence of novel and potentially successful viruses with distinct receptor usage and broader host tropism [16, 24, 60, 61]. de Klerk et al. (2022) [62] showed that recombination in coronaviruses is non-random, and the recombination breakpoints are conserved across multiple coronavirus subgenera, with hotspots of recombination within and near the spike gene. Consistent with their analysis, we also identified recombination breakpoints within the spike gene, highlighting the recombinant nature of the MERS-CoV, but also another bat merbecovirus’s (BtCoV-422), RBD region (Fig. 3).

The phylogenetic tree of the RBD shows that MERS-CoV groups with other known bat DPP4-using viruses, indicating that receptor acquisition likely took place in bats before its emergence in camels and/or any intermediate hosts. Since NeoCoV was sampled in bats [11], and all other known close MERS-CoV relatives circulate in bats [63], it is highly likely that the receptor switching recombination event generating MERS-CoV’s progenitor also took place in bats. However, the MERS-CoV spike-encoding region is still distinct to known DPP4-using bat viruses and clusters in the overall clade with fairly low bootstrap support (see Fig. 2B). Hence, it cannot be certain that the MERS-CoV spike came from a bat virus and not a yet undiscovered group of merbecoviruses circulating in a different reservoir host.

Although the MERS-CoV RBD is quite distant to that of all merbecoviruses sampled to date, the rest of the MERS-CoV genome is clearly nested within the ACE2-using group 1 clade, being relatively close to NeoCoV (Fig. 3). While it is well-established that MERS-CoV utilizes DPP4, it is alarming that a yet undiscovered close relative of MERS-CoV (i.e. split after the MERS-CoV – NeoCoV separation and before the recombination event leading to the present MERS-CoV clade) might still be circulating in the wild. It is worth mentioning that the known group 1 virus ACE2s (NeoCoV and PDF-2180) cannot efficiently mediate entry into cells expressing human ACE2 [16]. However, single, key amino acid substitutions in the bat merbecovirus RBDs can increase entry efficiency, previously seen with specific ACE2 binding of other bat coronaviruses [64]. There is increasing evidence that viral entry can be a rather transient barrier to virus spillover [65], hence the possibility of an ACE2-using MERS-CoV-like virus posing a significant zoonotic threat to humans should be considered. Additionally, hACE2 and hDPP4 are expected to differ in their tissue-specific expression patterns, suggesting that such a virus may have very different disease and transmission outcomes compared to MERS-CoV as we know it. This calls for systems-based research on the receptor usage preference of merbecoviruses to identify potential zoonotic risks posed by these viruses.

Albeit quite sparse, the geographical distribution of the known merbecoviruses could provide some helpful insights into the location of MERS-CoV’s proximal ancestor. The fact that both PDF-2180 and NeoCoV have been sampled in Africa, paired with the first identification of MERS-CoV in Jeddah (Saudi Arabia) (Fig. 4), suggests the potential circulation of MERS-CoV’s proximal, bat-infecting ancestors (before and after the RBD switch) somewhere in the African continent, followed by spillover to camels [11] and subsequent movement to Saudi Arabia. However, it is essential to note that this hypothesis is speculative as our understanding of bat merbecovirus ecology in Africa is still in its infancy [66]. All known bat DPP4-using merbecoviruses have been sampled in Southern China, but it is difficult to infer that receptor acquisition of MERS-CoV via recombination happened in China due to the distant location of the major parent of MERS-CoV (a virus most closely related to NeoCoV). Moreover, the recombination analysis could not identify the minor parent in the available virus dataset, indicating that the clade MERS-CoV acquired its RBD from is genetically distant to the viruses sampled so far. This further complicates the geographical inference of MERS-CoV’s origins which relies on the virus’s genetic relatedness to known genomes. Assuming that genetic distance partly translates to geographic distance, due to host movement and virus dispersal, this unsampled group of merbecoviruses may circulate in a distant location to that of the known DPP4-using viruses. Without comprehensive virus surveillance in African bats, determining the exact origin of MERS-CoV’s RBD remains a challenging task. Therefore, increased bat sampling in Africa, but also other regions where merbecoviruses have previously been detected (including Asia and Europe) is crucial for understanding MERS-CoV’s zoonotic origins and preventing future spillovers.

One of the limitations of this study is the lack of experimental evidence on the influence of recombination on the receptor switching events between MERS-CoV and its close relatives. Moreover, we failed to identify the minor recombination parent in the MERS-CoV’s RBD acquisition event and this is due to the limited number of *Merbecovirus* sequences publicly available at the time of this study. Despite the lack of a clear parental sequence in our dataset, this recombination event is confidently detected, being statistically supported by 7 out of the 7 independent recombination detection tools used here and validated through phylogenetic analyses. Further investigations, including intensive wildlife sampling efforts, are warranted to fully determine the influence of recombination on the receptor switching events among merbecoviruses and elucidating the exact steps that led to the emergence of MERS-CoV.

### Supplementary Information

**Additional file 1: Table S1.** Representative of merbecoviruses classified according to their strain name, GenBank accession number, host, country, group, and the representative sequences used in RDP5.

**Additional file 2: Table S2.** List of putative recombination events detected by at least six statistical methods implemented in RDP5.

**Additional file 3: Table S3.** One recombination event identified in the spike gene of HCoV-EMC/2012.

## Data Availability

The multiple sequence alignment, phylogenetic tree and code used in the present study are available in the following GitHub repository: https://github.com/TheSatoLab/Merbecovirus_recombination.
